# Effect of Resistance Training on Extracellular Matrix Adaptations in Skeletal Muscle of Older Rats

**DOI:** 10.3389/fphys.2018.00374

**Published:** 2018-04-11

**Authors:** Vinicius Guzzoni, Manoel B. T. Ribeiro, Gisele N. Lopes, Rita de Cássia Marqueti, Rosângela V. de Andrade, Heloisa S. Selistre-de-Araujo, João L. Q. Durigan

**Affiliations:** ^1^University of Brasília, Brasília, Brazil; ^2^Department of Physical Education, University of Brasília, Brasília, Brazil; ^3^Department of Physiological Sciences, Center of Biological and Health Sciences, Federal University of São Carlos, São Carlos, Brazil; ^4^Graduate Program of Rehabilitation Sciences, University of Brasilia, Brasilia, Brazil; ^5^Graduate Program of Genomics and Proteomics, Catholic University of Brasilia, Brasilia, Brazil

**Keywords:** resistance training, aging, skeletal muscle, connective tissue, gene expression

## Abstract

Accumulation of connective tissue, particularly extracellular matrix (ECM) proteins, has been observed in skeletal muscles with advancing age. Resistance training (RT) has been widely recommended to attenuate age-induced sarcopenia, even though its effects on the components that control ECM turnover in skeletal muscles remain to be elucidated. Thus, the aim of this study was to determine the effects of RT on connective tissue content and gene expression of key components of ECM in the skeletal muscles of aged rats. Young (3 mo.) and older (21 mo.) adult male *Wistar* rats were submitted to a RT protocol (ladder climbing with 65, 85, 95, and 100% load), 3 times a week for 12 weeks. Forty-eight hours post-training, the soleus (SOL) and gastrocnemius (GAS) muscles were dissected for histological and mRNA analysis. RT mitigated the age-associated increase of connective tissue content in both muscles, even though mRNA levels of COL-1 and−3 were elevated in older trained rats. Overall, RT significantly elevated the gene expression of key components of connective tissue deposition (TGFβ and CTGF; MMP-2 and-9; TIMP-1 and−2) in the GAS and SOL muscles of older rats. In conclusion, RT blunted the age-induced accumulation of connective tissue concomitant to the upregulation of genes related to synthesis and degradation of the ECM network in the SOL and GAS muscles of older rats. Although our findings indicate that RT plays a crucial role reducing connective tissue accumulation in aged hindlimb muscles, key components of ECM turnover were paradoxically elevated. The phenotypic responses induced by RT were not accompanied by the gene expression of those components related to ECM turnover.

## Introduction

Connective tissue surrounds skeletal muscle fibers and contributes to the protection of fibers against contractile damage (Kragstrup et al., 2011). However, abnormal ECM remodeling has been shown to affect the functional properties of skeletal muscles (Alnaqeeb et al., 1984; Kragstrup et al., 2011; Gillies et al., 2012). Accumulation of connective tissue, particularly proteins of the extracellular matrix (ECM), has been observed in skeletal muscles with advancing age (Alnaqeeb et al., 1984; Wood et al., 2014). Moreover, ECM consists essentially of collagen fibrils that provides a structural scaffold for the muscle tissue (Aumailley and Gayraud, 1998; Bonnans et al., 2014).

ECM is continually remodeled through processes of synthesis, degradation, and reassembly of the protein network (Lu et al., 2011). Degradation is orchestrated by a fine balance between the proteolytic activity of metalloproteinases (MMPs) and their endogenous tissue inhibitors of metalloproteinase (TIMPs) (Alameddine and Morgan, 2016). MMPs play a key role in ECM homeostasis which is supported by the direct cleavage of connective tissue collagen, activation of latent enzymes, and liberation of structural or bioactive molecules from the ECM (Alameddine and Morgan, 2016). Even though the proteolytic activity of gelatinases is essentially to degrade denatured collagens (Chen and Li, 2009), MMP-2 and MMP-9 have been shown to be involved in the degeneration and regeneration of skeletal muscles (Kherif et al., 1999; Fukushima et al., 2007). TIMP-1 and TIMP-2 each inhibit the activity of all MMPs with a preference for inhibiting MMP-2 and MMP-9, respectively (Kjær, 2004). In addition, TIMP-2 is involved in MMP-2 activation (Wang et al., 2000).

In addition to the remodeling process, ECM also functions as a ligand “reservoir” by binding growth factors, such as TGFβ and CTGF (Bonnans et al., 2014). TGFβ plays a pivotal role in the fibrotic process and in regulation of ECM production (Mendias et al., 2012). Moreover, TGFβ stimulates the expression of ECM-related genes, such as COL-I, COL-III, and TIMP-1 (Verrecchia et al., 2001). In this regard, CTGF represents a downstream regulatory molecule of TGFβ in the fibrogenesis process (Leask et al., 2002).

Recent evidence has shown the effects of aging and exercise training on skeletal muscle composition (Lacraz et al., 2015; Mikkelsen et al., 2017). Whereas, aging consistently leads to connective tissue accumulation in skeletal muscles (Mohan and Radha, 1980; Alnaqeeb et al., 1984; Zimmerman et al., 1993; Gosselin et al., 1998; Haus et al., 2007; Kragstrup et al., 2011), intracellular components of connective tissue deposition and ECM turnover accompanying the phenotype responses have not been clearly defined. Aging seems to induce collagen synthesis (Mays et al., 1991; Haus et al., 2007) and upregulates modulators of ECM synthesis, such as TGFβ and CTGF (Ihn, 2002; Mann et al., 2011; Dworatzek et al., 2015), and degradation (MMPs and TIMPs) (Dennis et al., 2008; Kwak et al., 2011; Yu et al., 2013). On the other hand, exercise training has been demonstrated to counteract the age-associated fibrosis in skeletal muscles (Zimmerman et al., 1993; Gosselin et al., 1998; Koskinen et al., 2002), even though its effects on those intracellular components still need more investigation. In fact, resistance training (RT) has been recommended to attenuate age-induced sarcopenia (Melov et al., 2007). Furthermore, RT has been shown to reduce collagen volume fraction in the human heart, demonstrating its clinical importance (Alves et al., 2014). Indeed, we have previously demonstrated that RT attenuated age-related biomechanical changes in the tendons of rats (de Cássia Marqueti et al., 2017). However, functional and structural differences have been observed between connective tissues of tendons and skeletal muscles (Kjær, 2004).

Considering that aging contributes to the atrophy of skeletal muscles by transcriptional mechanisms (Suetta et al., 2012), and mutations in gene encoding factors responsible for the connective tissue composition might lead to tissue defects (Bonnans et al., 2014), it is worthwhile to investigate whether RT modulates the gene expression of key components of connective homeostasis during aging. Thus, the aim of this study was to determine the effects of RT on connective tissue content and gene expression of key components related to connective tissue homeostasis in skeletal muscles of aged rats. We hypothesized that RT would attenuate the age-induced connective tissue accumulation in the skeletal muscles of older rats, concomitant to upregulation of modulators of ECM degradation (MMP-2/MMP-9; TIMP-1/TIMP-2) and downregulation of elements related to ECM synthesis (COL-1A1/COL-3A1; TGFβ and CTGF) at a transcriptional level.

## Materials and methods

### Experimental design

Young adult (3 mo.) and aged (21 mo.) male Wistar rats were housed in plastic cages under controlled environmental conditions (12-h light/dark cycle) with free access to water and standard chow (Socil, São Paulo). The University of São Carlos Ethics Committee approved the experimental procedures (number 059/2010) and the study was conducted in accordance with the National Guide for the Care and Use of Laboratory Animals (National Research Council, 1996). The rats were randomly distributed into four experimental groups: young sedentary (YS); young trained (YT); older sedentary (OS); and older trained (OT).

### Resistance training (RT) protocol

The adapted RT protocol (ladder climbing with 65, 85, 95, and 100% load), 3 times a week for 12 weeks was recently reported by our laboratory (de Cássia Marqueti et al., 2017).

Rats were adapted to climbing a ladder (1.1 m; 0.18 m, 2-cm grid, 80° incline) during 3 days with a load apparatus attached to their tails. They were initially placed at the bottom of the ladder and familiarized with climbing. During familiarization period, the maximal load carried was determined to begin the exercise training. RT protocol occurred in alternative days, 3 times per week, over a 12-week period. The length of the ladder required the animals to make 8–12 dynamic movements per climb, with 4–9 climbs/session. The climbs consisted of carrying a progressive load of 65, 85, 95, and 100% of the maximal carrying capacity of each animal. At the end of these 4 climbs, an additional 30-g weight was added to the load apparatus until the animal were not able to climb the entire ladder successfully. At the top of the ladder the rats rested for 2 min in a housing chamber. RT session consisted of 5–8 dynamic movements per climb over 6–8 s. The RT model was based on previously validated protocol (Hornberger and Farrar, 2004; Guzzoni et al., 2017).

### Muscle sample collection

After the experimental periods, the animals were weighed and euthanized. Then, the right soleus (SOL) and gastrocnemius (GAS) muscles were carefully dissected. The proximal fragment was used for the histological analysis and the distal portion for the mRNA analysis. For the histological analysis, the muscle fragment was immediately frozen in isopentane, pre-cooled in liquid nitrogen, and stored in a freezer at −80°C (Forma Scientific, Marietta, Ohio). For the mRNA analysis, the muscle fragment was frozen in liquid nitrogen and stored at −80°C.

### Trichrome masson staining

Briefly, 10 μm of frozen GAS and SOL cross-sections were cut at −16°C and placed on a slide. After a 20 min drying period, slides were placed in a Columbia jar and fixed overnight at room temperature in Bouin's solution. Slides were then rinsed in distilled water for 3 min, then running tap water for 5 min. Cross sections were then stained in Weigert's hematoxylin for 15 min, and washed in distilled water, then running tap water for 5 min. Muscle fibers were then stained with 1% Biebrich scarlet-acid fuschin for 15 min, then washed in distilled water for 5 min. After differentiation in 2.5% phosphomolybdic-phosphotungstic acid solution for 15 min, the sections were transferred directly into 2.5% aniline blue solution for 12 min. GAS and SOL sections (*n* = 6/group) were differentiated in 1% acetic acid solution for 3 min, dehydrated in 95 and 100% ethanol, then cleared in xylene. In this technique, skeletal muscle fibers were stained a bright red and connective tissue were stained a bold blue.

### Connective tissue quantification

Images of SOL and GAS muscles were randomly captured on a Zeiss Axioplot Vision-series microscope (Kim et al., 2008). All images were captured using 680 × 512-pixel resolution and quantified using the NIH Image J software (National Institutes of Health, Bethesda, MD). To quantify the cellular regions, images were converted to RGB stack. Connective tissue surrounding the skeletal muscle tissue was separated by manual threshold of hue (121–179), saturation (20–255), and brightness (10–255), as supported by others (Schneider et al., 2012; Bauman et al., 2014). Connective tissue content was quantified as mean blue intensity per tissue area and expressed as arbitrary unit (AU). Six images of the SOL and GAS muscles were randomly acquired from each experimental group (*n* = 6/group). Seven cells of each image, for a total of 42 muscle cells per animal, were acquired and measured at 400x magnification (Guzzoni et al., 2017). The identity of the samples was blinded to the examiner.

### RNA isolation and analysis

RNA was isolated using 1 ml of Trizol reagent (Invitrogen, Carlsbad, CA) according to the manufacturer's instructions. The extracted RNA was dissolved in hydroxymethyl-aminomethane·hydrochloride (tris-HCl) and ethylenediaminetetracetic acid (TE), pH 7.6, and quantified by spectrophotometry. The purity was assessed by determining the ratio of the absorbance at 260 and 280 nm. The integrity of the RNA was confirmed by inspection of ethidium bromide stained 18S and 28S ribosomal RNA under ultraviolet light. Total RNA was reverse transcribed into complementary deoxyribonucleic acid (cDNA).

### Analysis by quantitative polymerase chain reactions (qPCR)

Detection of mRNA for the different experimental and control samples were performed in a Rotor Gene 3000 (Cobert's, Sydney, Australia). The amplification mixes contained 1 μl of cDNA sample, 25 μl of SYBR Green fluorescent dye, Master mix (Applied Biosystems, Foster City, CA), and 180 nM of each primer in a final volume of 50 μl. Thermal cycling conditions included 10 min at 95°C, and then 40 cycles every 15 s at 94°C, 30 s at 48°C for MMP-2, MMP-9, TIMP-1, TIMP-2, and GAPDH, and at 48°C for COL-I, COL-III, TGF-β, and CTGF, respectively, and then 1 min at 72°C, and finally 10 min at 72°C. For each gene, all samples were amplified simultaneously in duplicate in one assay run. Data were analyzed using the comparative cycle threshold (Ct) method according to the manufacturer's guidelines (Bulletin No. 2, Applied Biosystems). The list of oligonucleotide primers are shown in Table 1. The GAPDH mRNA was used as an internal control (Dheda et al., 2004).

**Table 1:** List of oligonucleotide primers.

	**Forward**	**Reverse**
COL1A1	ATCAGCCCAAACCCCAAGGAGA	CGCAGGAAGGTCAGCTGGATAG
COL3A1	TGATGGGATCCAATGAGGGAGA	GAGTCTCATGGCCTTGCGTGTTT
TGF-β1	CCCCTGGAAAGGGCTCAACAC	TCCAACCCAGGTCCTTCCTAAAGTC
CTGF	CAGGCTGGAGAAGCAGAGTCGT	CTGGTGCAGCCAGAAAGCTCAA
MMP-2	CTGGGTTTACCCCCTGATGTCC	AACCGGGGTCCATTTTCTTCTTT
MMP-9	GGATGTTTTTGATGCCATTGCTG	CCACGTGCGGGCAATAAGAAAG
TIMP-1	ATAGTGCTGGCTGTGGGGTGTG	TGATCGCTCTGGTAGCCCTTCTC
TIMP-2	GGACACGCTTAGCATCACCCAGA	GTCCATCCAGAGGCACTCATCC
GAPDH	CCACCAACTGCTTAGCACC	GCCAAATTCGTTGTCATACC

*COL-I, type-I collagen; COL-III, type-III collagen; TGF-β1, transforming growth factor beta-1; CTGF, connective tissue growth factor; MMP-2, metalloproteinase 2; MMP-9, metalloproteinase 9 TIMP-1, tissue inhibitor of metalloproteinases-1; TIMP-2, tissue inhibitor of metalloproteinases-2; GAPDH, glyceraldehyde 3-phosphate dehydrogenase*.

### Statistical analysis

The Shapiro-Wilk and Levene's tests were used to investigate whether the data were normally distributed. As all included variables were normally distributed, a two-way ANOVA (aging and exercise training as factors) followed by a Tukey HSD *post-hoc* test. Differences were considered significant when *p* < 0.05. Statistical analysis was performed using the GraphPad Prism 7.0c software.

## Results

**Figure 1:**
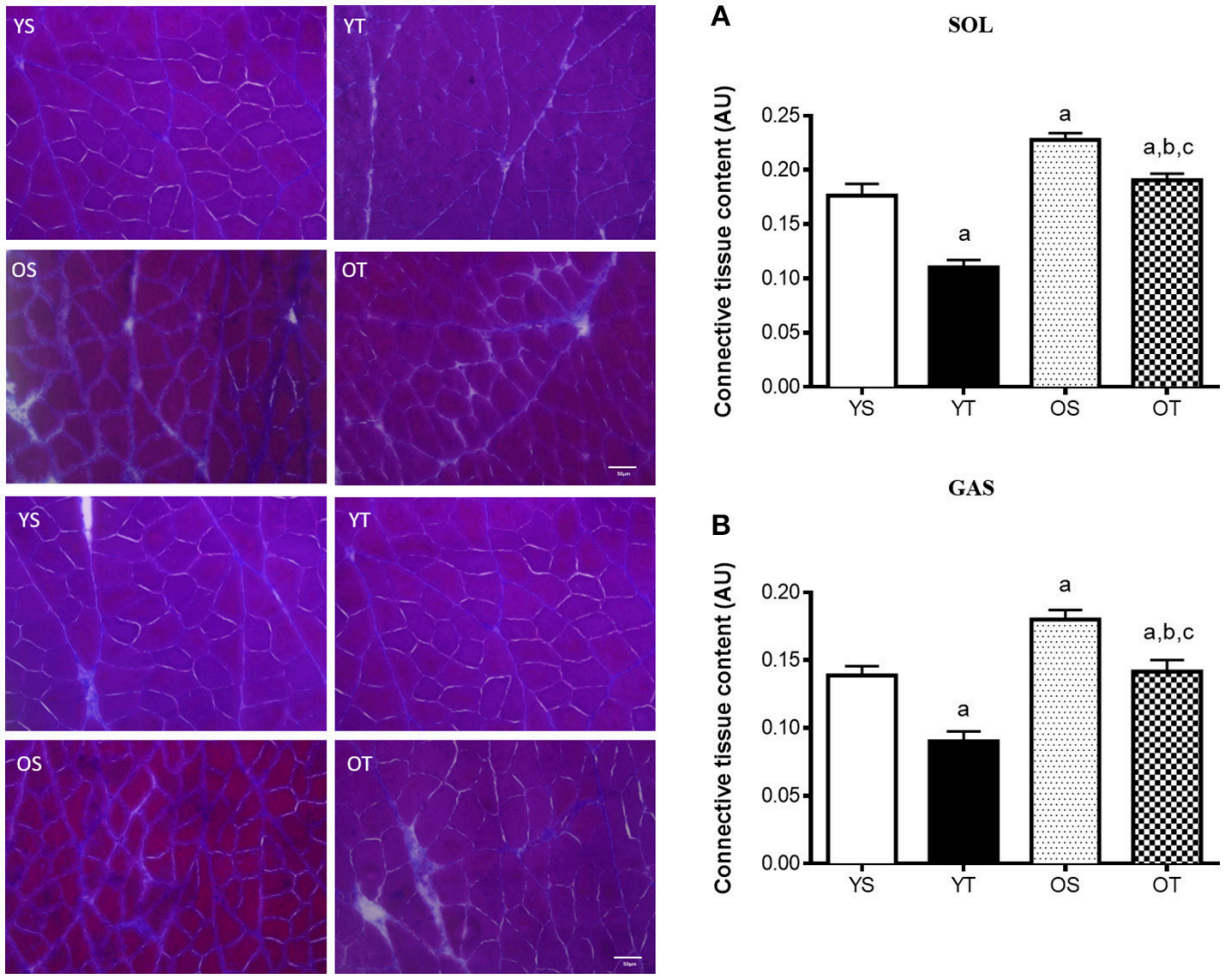
Light microscope images of Masson's Trichrome and quantification of connective tissue in SOL **(A)** and GAS **(B)** muscles, at 400X magnification. Quantification of connective tissue content was expressed as arbitrary unit (AU). Groups: young sedentary (YS), young trained (YT), older sedentary (OS), and older trained (OT) rats. Values are expressed as means ± SEM. Two-way ANOVA, *p* < 0.05: ^a^vs. YS; ^b^vs. YT; ^c^vs. OS. *n* = 6/group.

**Figure 2:**
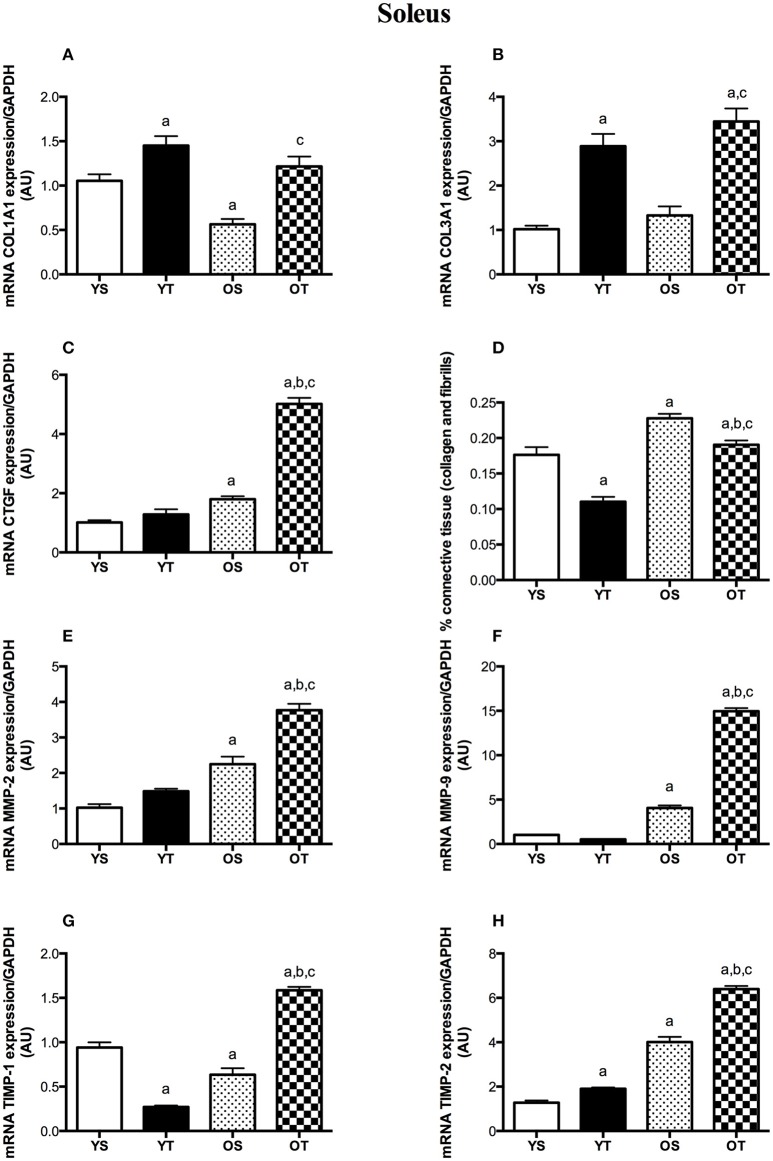
mRNA levels of COL1A1 **(A)**, COL31A1 **(B)**, CTGF **(C)**, TGF-β **(D)**, MMP-2 **(E)**, MMP-9 **(F)**, TIMP-1 **(G)**, and TIMP-2 **(H)** of SOL muscle. Groups: young sedentary (YS), young trained (YT), older sedentary (OS), and older trained (OT). Values are expressed as means ± SEM. Two-way ANOVA, *p* < 0.05: ^a^vs. YS; ^b^vs. YT; ^c^vs. OS. *n* = 6/group.

RT mitigated the age-associated increase of connective tissue content both in the SOL and the GAS muscles (OT vs. OS). RT also attenuated the accumulation of connective tissue in both muscles of young rats (YT vs. YS) (Figures 1A,B). In the SOL muscle, RT elevated mRNAs levels of COL1A1 and COL3A1 in older rats (OT vs. OS) (Figures 2A,B). RT led to marked increases in COL-3A1, TGF-β, CTGF, MMP-2, MMP-9, TIMP-1, and TIMP-2 transcripts when compared with their age-matched counterparts (OT vs. YS; OT vs. OS) (Figures 2C–H). While COL3A1 was unchanged, COL1A1 transcript was lower in the SOL muscle of OS rats than in the YS group, even though TGF-β and CTGF transcripts increased in OS rats when compared with the YS group (Figures 2A–D). Whereas, aging also elevated mRNA levels of MMP-2, MMP-9, and TIMP-2 transcripts, TIMP-1 was reduced in OS rats when compared with the YS group (Figures 2E–H). In the SOL muscle of both young groups, mRNA levels of COL1A1 and COL3A1 were dramatically elevated in YT rats in relation to YS rats (Figures 2A,B). Likewise, TGF-β mRNA levels increased in YT rats in comparison to the YS group (Figure 2C). Whereas, RT evoked a reduction in TIMP-1 mRNA levels, TIMP-2 transcript levels were greater in YT rats than in YS rats (YT vs. YS) (Figures 2G,H).

**Figure 3:**
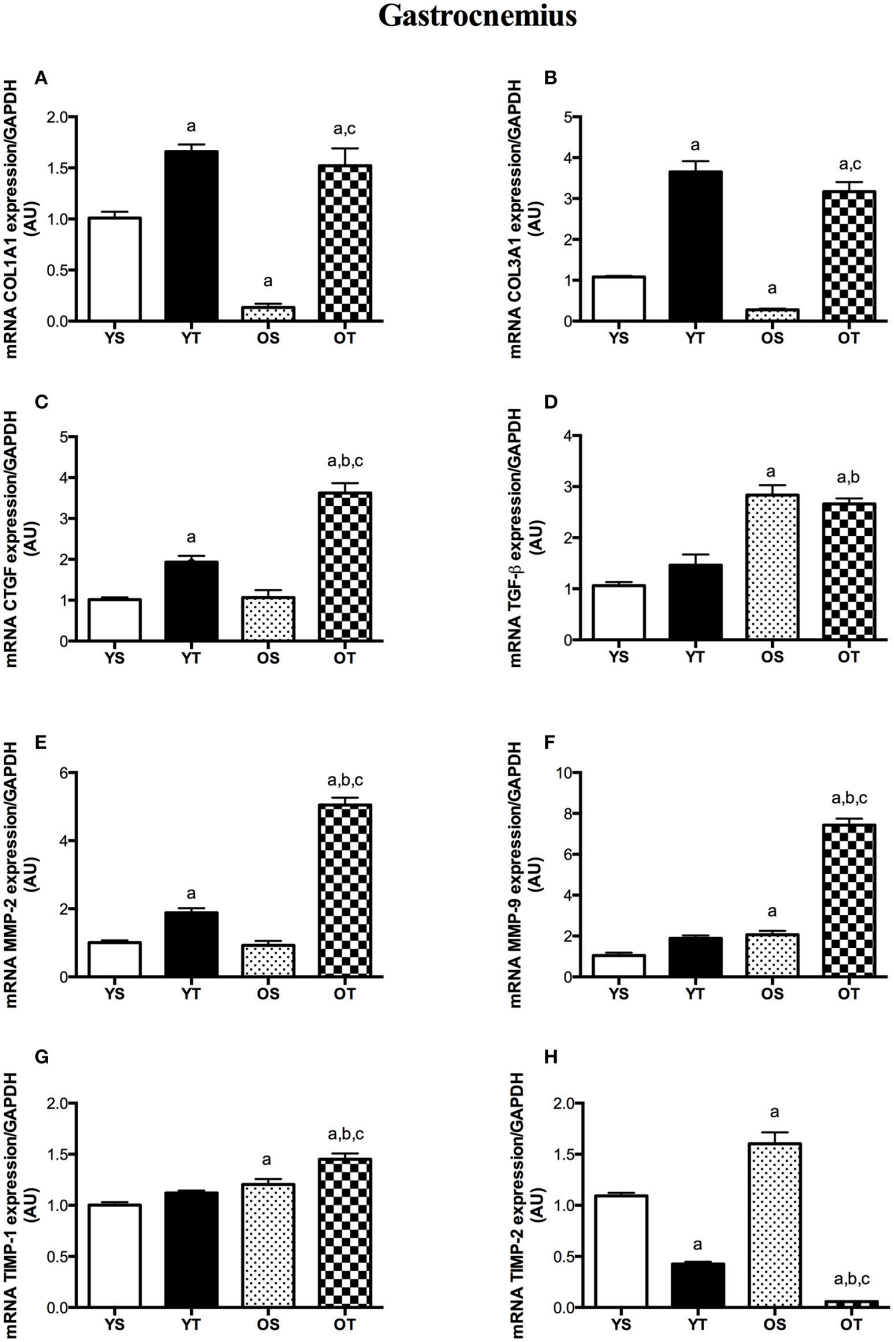
mRNA levels of COL1A1 **(A)**, COL31A1 **(B)**, CTGF **(C)**, TGF-β **(D)**, MMP-2 **(E)**, MMP-9 **(F)**, TIMP-1 **(G)**, and TIMP-2 **(H)** of GAS muscle. Groups: young sedentary (YS), young trained (YT), older sedentary (OS), and older trained (OT). Values are expressed as means ± SEM. Two-way ANOVA, *p* < 0.05: ^a^vs. YS; ^b^vs. YT; ^c^vs. OS. *n* = 6/group.

In the GAS muscle, RT also induced substantial increases in mRNA levels of COL1A1, COL3A1, and CTGF when compared with YS and OS rats (OT vs. OS) (Figures 3A,B,D). TGF-β transcript was greater in OT than in YS rats (Figure 3C). While COL1A1 and COL3A1 transcripts were lower in the GAS muscle of OS rats, TGF-β mRNA levels significantly increased with aging (OS vs. YS and OT vs. YT) (Figures 3A–C). Transcripts of MMP-2, MMP-9, and TIMP-1 were higher in OT rats than the rest of experimental groups (OT vs. YS; OT vs. YT; OT vs. OS) (Figures 3E–G). Conversely, RT resulted in a marked reduction of TIMP-2 transcript in older rats when compared with other groups (OT vs. YS; OT vs. YT; OT vs. OS) (Figure 3H). MMP-9, TIMP-1, and TIMP-2 mRNA levels increased with aging (OS vs. YS) (Figures 3F–H). In the GAS muscle of young rats, mRNAs levels of COL1A1 and COL3A1 were elevated in YT rats when compared with those transcripts in YS rats (Figures 3A,B). Likewise, CTGF mRNA levels also increased in YT rats in relation to YS group (Figure 3D). While RT elevated MMP-2 transcript, TIMP-2 was reduced in the GAS muscle of YT rats when those were compared with their respective YS rats (Figures 3E,H).

## Discussion

The novel insight of this study relies on the upregulation of key components of connective tissue at a transcriptional level in skeletal muscles of older rats submitted to RT, concomitant to the reduction of connective tissue accumulation. RT blunted the age-induced accumulation of connective tissue concomitant to the upregulation of genes related to synthesis (COL-1A1/COL-3A1; TGFβ and CTGF) and degradation (MMP-2/MMP-9; TIMP-1/TIMP-2) of the ECM network in the SOL and GAS muscles of older rats. Our findings indicate that RT plays a crucial role against connective tissue accumulation in aged hindlimb muscles, even though gene expression of key components of the connective tissue was not accompanied by the phenotypic responses induced by resistance training.

In fact, we clearly observed increases in connective tissue levels in SOL and GAS of older rats, which are in agreement with previous studies (Mohan and Radha, 1980; Alnaqeeb et al., 1984; Gosselin et al., 1998). According to Lacraz et al. (2015), skeletal muscle aging is associated with a decrease of regenerative potential due to the loss of function of myogenic progenitor cells (MPCs). The deposition of ECM in aged skeletal muscles can influence the biomechanical properties of myofibers by increasing their stiffness, which directly impacts MPC function (Lacraz et al., 2015). The current study suggested that the differential level of collagen accumulation might be related to the aging-associated muscle loss. However, collagen content in skeletal muscles from humans was unchanged, even though cross-links of collagen fibrils and advanced glycation end products (AGEs) were increased with aging (Haus et al., 2007). Although endurance training had no effect on ECM content in the SOL muscle from older rats, age-induced increases of collagen cross-links were attenuated by training (Zimmerman et al., 1993; Gosselin et al., 1998). Moreover, jump training has been shown to elevate collagen concentration in hindlimb muscles from rabbits (Ducomps et al., 2003). On the other hand, we observed that RT (ladder climbing exercise) alleviated the age-induced accumulation of connective tissue in SOL and GAS muscles, suggesting that resistance training plays a role in age-associated connective tissue deposition in slow and fast twitch fibers (predominantly in SOL and GAS muscles, respectively). On this basis, we have recently demonstrated that the same RT model alleviated the age-induced reduction in CSA of SOL and GAS muscles, which was linked to the upregulation of pro-growth molecules, including IGF-1, mTOR, p70S6K-1, and MyoD (Ribeiro et al., 2017). Furthermore, we observed that RT protocol attenuated the age-related weight loss in the same rats used in this study, suggesting its role both in the whole-body homeostasis and in the muscles adaptations (Ribeiro et al., 2017). Of note, the discrepancies among the studies might be explained by the changes within ECM network across different tissues and species (Kular et al., 2014).

Collagen synthesis has been shown to be active with advancing age (Mays et al., 1991; Babraj et al., 2005). However, we observed reduced mRNA levels of both collagens (I and III) in the GAS muscle from older rats (OS vs. YS), even though COL-III was unchanged in the SOL muscle with aging. In agreement with our findings, aging has been shown to reduce pro-collagen type I gene expression in skeletal muscles (Mamuya et al., 1992; Goldspink et al., 1994; Pattison et al., 2003). Our findings suggest post-transcriptional regulation rather than a transcription mechanism. Also, connective tissue accumulation observed in the SOL and GAS of OS rats might reflect a decreased degradation rather than increased synthesis.

In contrast to aging effects, upregulation of COL-I and COL-III mRNA levels was observed after RT, both in SOL and GAS muscles, suggesting that exercise training modulates collagen synthesis in skeletal muscles at a transcriptional level. In accordance with our findings, upregulation of COL-I and COL-III genes was observed in the soleus and quadriceps femoris muscles after running exercise in rats, although protein levels were unchanged (Han et al., 1999). Similar results were documented in a previous study using resistance exercise (Holm et al., 2010). Thus, given that mechanical loading might trigger collagen synthesis in skeletal muscles (Kjær, 2004), we suggest that RT training plays a substantial role in collagen metabolism at a pre-translational mechanism level. Furthermore, increased rates of collagen synthesis may be associated with the repair process of tissue damage following exercise training (Koskinen et al., 2001a; Close et al., 2005).

TGFβ plays a pivotal role in age-induced fibrosis (Mann et al., 2011). Indeed, we found a marked increase in TGFβ mRNA levels in SOL and GAS muscles with aging (OS *vs*. YS), which might be associated with increased connective tissue content observed in both muscles from OS rats. Our findings are in accordance with studies that demonstrated increased protein levels of TGFβ in hearts (Kwak et al., 2011; Dworatzek et al., 2015) and mRNA expression in kidneys (Ruiztorres et al., 1998) of old rats. Interestingly, CTGF has been suggested to be involved in fibrosis (Ihn, 2002; Lipson et al., 2012). CTGF synthesis is stimulated by TGFβ (Chen et al., 2000; Kjær, 2004). With advancing age, CTGF mRNA expression was elevated in the hearts from rats (Wang et al., 2010). However, there is limited understanding of CTGF expression in aged skeletal muscle. In the SOL muscle, we observed higher CTGF transcripts in OS rats but not in the GAS muscle (OS vs. YS). Although aging reduced either COL-I synthesis in the SOL muscle or COL-I and COL-III mRNA levels in the GAS, we might speculate that aging modulates the intracellular signaling of fibrosis at a transcriptional level, even considering that CTGF mRNA levels were unchanged in the GAS muscle of OS rats.

Mechanical loading activates gene expression of CTGF in fibroblasts, which induces the accumulation of collagen I synthesis and matrix proteins (Schild and Trueb, 2002). In fact, RT led to substantial increases in both TGF-β and CTGF transcripts of SOL and GAS muscles, which might be linked to the increases of COL-I and COL-III synthesis in both muscles of OT rats. Thus, we suggest that RT, as a mechanical stress condition, might modulate cell signaling of muscle fibrosis in a transcriptional manner. Our findings are supported by studies that observed increases of TGFβ synthesis in different cell types (Breen et al., 1996; Cillo et al., 2000; Skutek et al., 2001), which could indicate the release of TGFβ from tissues mechanically activated during exercise training (Kjær, 2004).

Given that connective tissue content was affected by aging and RT, we hypothesized that MMP synthesis would be modulated by those factors. Both gelatinases, MMP-2 and−9, degrade denatured collagens and exhibit specificity for native collagen types (Aimes and Quigley, 1995; Mackey et al., 2004; Bigg et al., 2007). MMP-2 is synthesized by fibroblasts and has been associated with myofiber regeneration while MMP-9, produced by leukocytes, is involved in the inflammatory response (Kherif et al., 1999). In this study, MMP-9 transcript was elevated in both muscles, while MMP-2 mRNA levels were only increased in the SOL muscle of OS rats. Increased MMP-2 and−9 expressions have been shown to be increased with aging (Yu et al., 2013) while lower levels of MMP-2 gene expression were observed in aged skeletal muscles (Dennis et al., 2008), even though there is no evidence of MMP-9. To the best of our knowledge, this is the first time that both gelatinases (MMP-2 and MMP-9) were demonstrated to be modulated by aging in skeletal muscles of rats.

Endurance exercise has been demonstrated to increase MMP expression in skeletal muscles (Rullman et al., 2009). On the other hand, we found substantial increases in MMP-2 and MMP-9 transcripts in both muscles of older trained rats (OT vs. OS). Indeed, exercise training has been shown to modulate MMP expression, although the results are inconsistent (Jaoude and Koh, 2016). Our findings are in agreement with others, at least in part, in which exercise training elevated gene expression and activity of gelatinases (MMP-2 and−9) in skeletal muscles of rats (Koskinen et al., 2001b; Souza et al., 2014) and humans (Rullman et al., 2009). Given that increased transcripts of MMP-2 and MMP-9 were observed in both muscles of OT rats, accompanied by the attenuation of age-induced connective tissue accumulation, we might speculate that ECM undergoes remodeling by the MMP pathway following exercise-induced muscle damage, as has been documented by others (Mackey et al., 2004). However, our findings do not rely on a mechanistic approach, once the mRNA levels of MMPs were analyzed.

Considering that TIMPs contribute to the accumulation of connective tissue via MMP inhibition (Jaoude and Koh, 2016), concomitant to stimulation of TIMPs by a TGF-β-mediated mechanism (Edwards et al., 1987), we also evaluated their gene expression in both muscles. While TIMP-1 transcript was reduced in the SOL muscle with aging, OS rats showed increased mRNA levels of TIMP-1 in the GAS muscle (vs. YS). On the other hand, TIMP-2 mRNA levels increased in both muscles of OS rats. In fact, little evidence regarding the gene expression of TIMPs has been demonstrated in aged skeletal muscles. However, TIMP-1 and−2 protein levels were increased in the aging hearts of rats (Kwak et al., 2011).

TIMPs are also activated in response to physical activity (Koskinen et al., 2002). In fact, we observed greater mRNA levels of TIMP-1 in OT rats (vs. OS), even though TIMP-1 was reduced or unchanged in the SOL and GAS muscles of YT rats, respectively. However, TIMP-2 transcript was markedly reduced in the GAS muscle of OT rats, suggesting that TIMP-2 modulation would depend on the fiber type. Regardless of the inconsistency of results, our findings related to TIMP upregulation after training are in agreement with those of others (Koskinen et al., 2001b; Rullman et al., 2009; Scheede-Bergdahl et al., 2014).

Finally, it is important to emphasize that few studies are available about age-related changes in ECM remodeling regarding the SOL and GAS muscles. Considering the contrasting differences in fiber type composition, i.e., the SOL is predominately composed of a low percentage of fast-twitch fibers, whereas the GAS is predominately composed of a high percentage of fast-twitch fibers (Armstrong and Phelps, 1984; Staron et al., 1999), we choose the GAS and SOL muscles in order to investigate the effects of RT on different fiber types. Moreover, ladder climbing exercise has been shown to affect other muscles (Klitgaard, 1988; Duncan et al., 1998; Hornberger and Farrar, 2004), whereas the effect of this RT protocol on age-induced changes in the SOL and GAS muscles are scarce. Although fiber composition seems not to be affected by aging and RT in this study, further studies are warranted to evaluate whether cross-talk takes place between the intracellular pathways in response to RT and aging.

## Limitations

As a limitation of this study, hydroxyproline content analysis was not performed in this study. In fact, hydroxyproline content is well-established technique to determine collagen concentration, even though might be considered an indirect method for measuring collagen content, at least in part, in studies using heart tissue (Badenhorst et al., 2003).

## Conclusion

RT blunted the age-induced accumulation of connective tissue concomitant to the upregulation of genes related to the synthesis (COL-1A1/COL-3A1; TGFβ and CTGF) and degradation (MMP-2/MMP-9; TIMP-1/TIMP-2) of the ECM network in the SOL and GAS muscles of older rats. Our findings might represent a useful approach for clinical implications of RT in the elderly population.

## Author contributions

VG, MR, and GL performed the experiments as well as analyzed the data. VG, RdC, and JD wrote the manuscript. RdC, RdA, HS-d-A, and JD designed the work, analyzed data, and contributed to the writing of the manuscript.

### Conflict of interest statement

The authors declare that the research was conducted in the absence of any commercial or financial relationships that could be construed as a potential conflict of interest.
